# Palinacousis in amyloidosis: exploring the hallucinatory phenomenon in brain pathology—a case report

**DOI:** 10.1186/s13256-024-04575-3

**Published:** 2024-07-16

**Authors:** João Martins-Correia, Luísa Sousa

**Affiliations:** 1 Department of Public Health and Forensic Sciences, and Medical Education, Faculty of Medicine, University of Porto, Porto, Portugal; 2 UMIB, Unit for Multidisciplinary Research in Biomedicine, ICBAS, School of Medicine and Biomedical Sciences, University of Porto, Porto, Portugal; 3 Laboratory for Integrative and Translational Research in Population Health, ITR, Porto, Portugal; 4 Department of Neurology, Centro Hospitalar de Entre o Douro e Vouga, Santa Maria da Feira, Portugal; 5 Unidade Corino de Andrade, Department of Neurosciences, Centro Hospitalar Universitário do Porto, Porto, Portugal

**Keywords:** Amyloidosis, Palinacousis, Neuropsychiatry, Case report

## Abstract

**Background:**

Hereditary transthyretin amyloidosis, caused by transthyretin gene mutations, progresses with systemic impact and often presents peripheral neuropathy. Recent research reveals central nervous system involvement, marked by leptomeningeal amyloid accumulation and transient focal neurological episodes displaying cortical dysfunction.

**Case presentation:**

A 47-year-old Caucasian man with hereditary transthyretin amyloidosis presented with motor aphasia, right hemiparesis, fever, and an altered state of consciousness. Tests ruled out stroke or infection. While improving, the patient reported an ongoing auditory repetition phenomenon for 48 hours despite efforts to shift focus or introduce new stimuli.

**Conclusion:**

This represents the first known case report documenting palinacousis in hereditary transthyretin amyloidosis attributed to central nervous system involvement. This case highlights the complexities in assessment and management of patients when neurological and psychiatric symptoms overlap.

## Introduction

Hereditary transthyretin (ATTRv) amyloidosis is an autosomal dominantly inherited systemic disorder arising from mutations within the transthyretin (*TTR*) gene. Abnormal TTR forms amyloid deposits that accumulate systemically, including in the peripheral nervous system and the heart [1]. This rare and clinically heterogeneous condition, which is endemic to a limited number of geographical regions, including Portugal, Sweden, and Japan, typically begins as a peripheral sensory-motor neuropathy [2]. Historically, liver transplantation was the primary therapeutic intervention for ATTRv amyloidosis, since the liver is the main site of TTR production. However, the incidence of this procedure has decreased in recent years with the emergence of other disease-modifying therapeutics, such as TTR stabilizers and gene therapies [3].

Recent findings indicate that TTR amyloid deposition also occurs in the central nervous system (CNS), particularly on leptomeningeal surfaces and in arteries, leading to clinical manifestations after at least a decade of peripheral symptoms [4]. The most common manifestation of CNS involvement is transient focal neurological episodes (TFNEs), observed in up to 31% of patients with longstanding disease [5]. These episodes are also known as amyloid spells. They are characteristically brief, self-contained, and stereotypical episodes of focal cortical dysfunction [5]. Other manifestations include seizures, brain hemorrhages, and cognitive deficits [2].

## Case presentation

A 47-year-old Caucasian man with a history of ATTRv amyloidosis who underwent liver transplantation in 2003 and has been on cyclosporine therapy (50 mg, three times daily) presented to the emergency room with sudden onset of motor aphasia, right hemiparesis, fever, and an altered state of consciousness.

The patient’s condition, resulting from a Val30Met mutation in the *TTR* gene, began at age 20 years and progressively led to distal tetraparesis and anesthesia affecting the lower limbs, trunk, and distal segments of the upper limbs due to severe polyneuropathy. Additionally, infiltrative cardiomyopathy with cardiac conduction disturbances required the implantation of a pacemaker. Over the past 6 years, the patient experienced recurring episodes of transient focal deficits characterized by language impairment, each lasting no longer than 1 hour and resolving spontaneously, consistent with TFNEs.

In the emergency room, the possibility of a stroke was deemed unlikely, considering the patient’s medical history and the absence of abnormalities in cerebral computed tomography (CT) and angio-CT scans. An infectious cause was ruled out through cerebrospinal fluid analysis, which showed a white blood cell count of 3/µL, protein concentration of 102 mg/dL, and glucose concentration of 57 mg/dL. Bacterial, viral, and fungal cultures returned negative results. A cerebral magnetic resonance imaging (MRI) was not feasible owing to the patient’s incompatible pacemaker. A follow-up CT scan, conducted 24 hours after admission, did not reveal any new findings. An electroencephalogram (EEG) revealed slow, intermittent wave activity, primarily situated in the left anterior temporal area.

As the patient’s motor aphasia gradually improved, he reported an auditory phenomenon experienced within the initial 48 hours after symptom onset. This involved continuous repetition of a perceived sound stimulus. According to the patient, this repetition occurred regardless of the type of auditory stimulus and was limited to the last word heard in verbal speech. The characteristics of this auditory phenomenon were indistinguishable from other external sounds. Introducing a new stimulus or attempting to shift auditory focus could mitigate the echoing of the sound. After the first 48 hours of admission, there was a complete remission of symptoms.

## Discussion

The described auditory phenomenon is known as palinacousis. Information regarding this highly unusual condition is limited and primarily derived from a sparse number of case reports. Palinacousis, also termed auditory perseveration, represents an acquired auditory perceptual disorder characterized by the internal perseveration of an external auditory stimulus in a paroxysmal manner [6]. Although palinacousis was first described in 1971 [7], it remains a rare and complex condition that may be mistaken for certain psychiatric manifestations, especially in cases with a past history of psychiatric disorders. It is presumed to stem from cortical dysfunction, commonly observed in the temporal lobes, with a higher incidence on the left side. Consequently, the majority of published cases are reported in association with epileptic events [8–11], intracranial metastases [12], multiple sclerosis [13], and vascular lesions [14–16]. This aligns with the CNS involvement potentially observed in ATTRv amyloidosis, as illustrated in the presented case. The speculated causes include epileptiform activity and hyperexcitability of the auditory cortex [8]. However, the pathophysiology of palinacousis remains poorly understood [14]. In this case, while the presentation of palinacousis is associated with an underlying organic condition, subjectively, this experience closely resembles purely psychiatric auditory hallucinations. Despite its vivid tones, the patient maintained insight into its occurrence, and the auditory phenomenon could be extinguished by introducing a different stimulus.

Unlike all previously documented cases in the literature, this instance represents the first occurrence of palinacousis within the context of amyloid spells. This unique perspective offers an examination of this primarily neurological condition through a psychiatric lens. It emphasizes the necessity for interdisciplinary collaboration in diagnosing complex neurological conditions that may mimic psychiatric disorders, prompting a reconsideration of diagnostic boundaries. Furthermore, this case sparks a discourse on distinguishing between organic and psychiatric manifestations, highlighting the significance of a thorough clinical evaluation and the use of advanced diagnostic tools.

## Conclusion

This case report details a patient with ATTRv amyloidosis who presented with palinacousis, likely indicating CNS involvement. The distinction between neurological and psychiatric conditions can often be challenging, complicating the assessment and accurate management of situations where the two appear to overlap.

## Data Availability

Not applicable.

## Abbreviations

ATTRv: Hereditary transthyretin
CNS: Central nervous system
CT: Computed tomography
EEG: Electroencephalogram
MRI: Magnetic resonance imaging
TFNEs: Transient focal neurological episodes
TTR: Transthyretin
